# High-sensitivity C-reactive protein could be a potential indicator of bone mineral density in adolescents aged 10–20 years

**DOI:** 10.1038/s41598-022-11209-5

**Published:** 2022-05-03

**Authors:** Weiran Ye, Shi Cheng, Jin Xiao, Hui Yu

**Affiliations:** 1 Department of Endocrinology, South China Hospital, Health Science Center, Shenzhen University, Shenzhen, 518116 People’s Republic of China; 2 Department of Orthopedics, Guangdong Provincial People’s Hospital, Guangdong Academy of Medical Sciences, Guangzhou, 510080 People’s Republic of China

**Keywords:** Predictive markers, Bone, Osteoporosis

## Abstract

There was very limited evidence linking high-sensitivity C-reactive protein (HS-CRP) and total bone mineral density (BMD) in adolescents. The aim of this population-based study was to investigate the relationship between HS-CRP and total BMD in adolescents aged 10–20 years. A cross-sectional study was performed in the normal U.S. population from the data of the National Health and Nutrition Examination Survey (NHANES). The correlation between HS-CRP and total BMD was evaluated by using weighted multivariate linear regression models. And further subgroup analysis was conducted. There were 1747 participants in this study, 47.1% were female, 29.4% were white, 19.5% were black, and 22.3% were Mexican–American. In the multi-regression model that after the potential confounders had been adjusted, HS-CRP was negatively associated with total BMD. The negative association was also observed in the subgroup analyses stratified by gender and age. Our results demonstrated that higher HS-CRP was negatively correlated with total BMD in 10–20 years old adolescents.

## Introduction

During adolescence, especially before the age of 20 years, bone accumulation and growth are rapid^1^. The acquisition of BMD during this period is essential for bone accumulation and bone formation, in order to obtain a greater peak bone mass, thereby preventing osteoporosis in older age^2–4^. In a sense, osteoporosis has also been considered to be an adolescent disease.

The clinical assessment of risk factors related to osteoporosis is helpful for the early diagnosis, prevention and management of osteoporosis. Therefore, some less studied or new biomarkers have been given more attention for their relevance to bone health, such as C-reactive protein (CRP). Previous animal studies have shown that inflammatory markers such as CRP are significantly associated with increased bone resorption and decreased bone formation^5,6^. Moreover, epidemiologic studies have proved that serum CRP levels are negatively associated with BMD^7–9^. Thus HS-CRP as a more precise measure than traditional CRP is likely to be a potential indicator of BMD. However, there is no evidence linking HS-CRP and BMD in adolescents till now. Herein, based on the 2015–2018 National Health and Nutrition Examination Surveys (NHANES), we conducted a cross-sectional study with a large sample to investigate the association of HS-CRP with BMD in adolescents aged 10–20 years.

## Materials and methods

### Study population

All analyzed data in this study was extracted from the NHANES. The NHANES is a complex and multi-stage probability sample of the civilian noninstitutionalized population in the United States. These cross-sectional investigations were carried out by the National Centre for Health Statistics (NCHS). The detail information about the NHANES methodology could be found at the website: www.cdc.gov/nchs/nhanes/. For this study, we used data from the 2015 to 2018 survey cycles, when HS-CRP was available.

**Figure 1:**
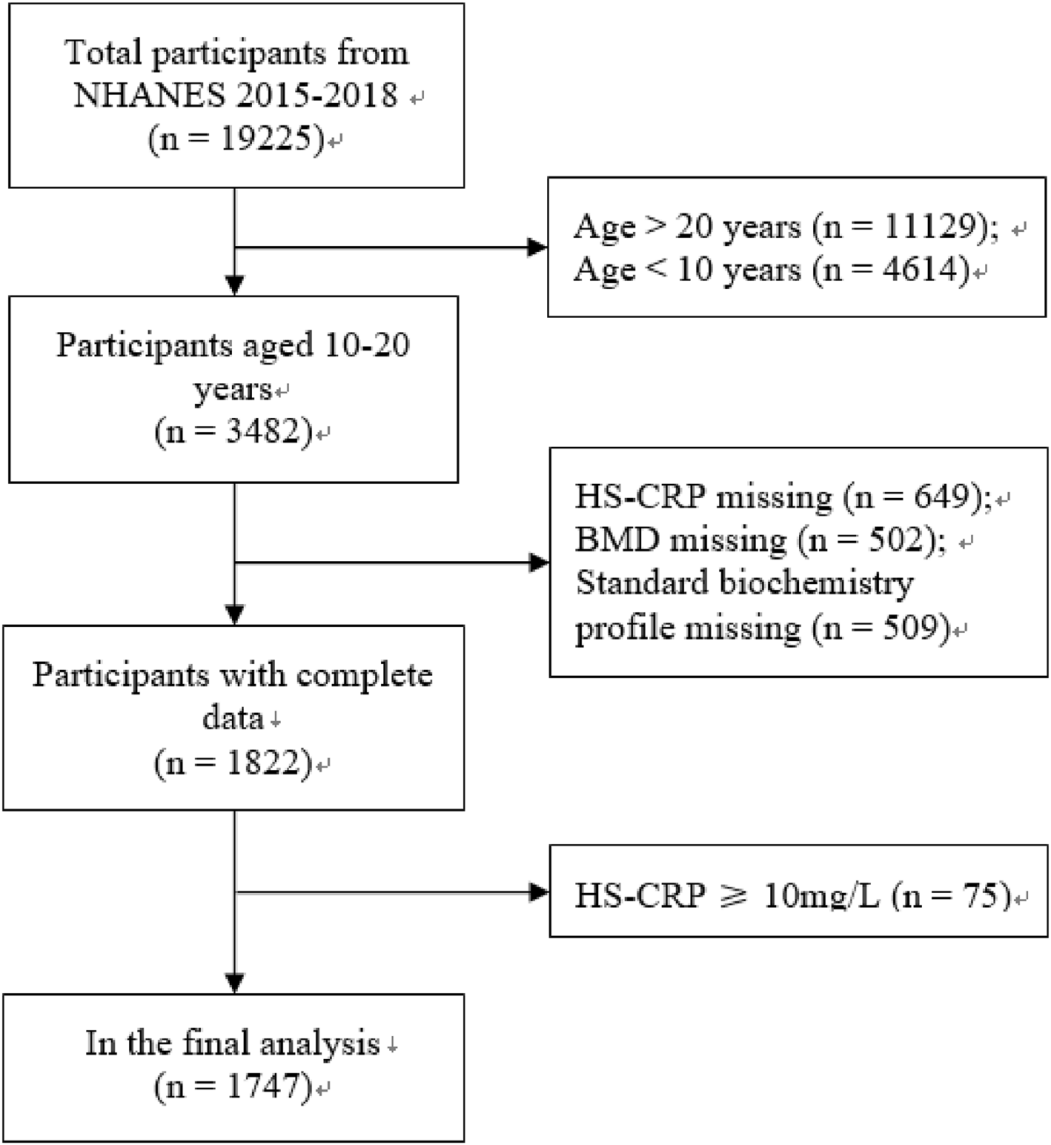
Flow chart of sample selection.

The inclusion of participants was limited to age 10–20 years (n = 3482). 649 participants were excluded for HS-CRP missing, and 502 participants were excluded for BMD missing. 509 participants were excluded for missing all blood biochemical data. Considering that HS-CRP ≥ 10 mg/L was an abnormal stress state^10^, 75 subjects were excluded. Finally, 1747 participants were included in the analysis. The flow chart of sample selection was shown in Fig. 1. The survey program was approved by the NCHS Institutional Review Board, and all participants had provided informed consent on their own or legally authorized representatives^11^. All methods were in accordance with the guidelines and regulations of the NCHS Institutional Review Board.

### Covariates

HS-CRP (independent variable) and BMD (dependent variable) were the main variables of this study. The HS-CRP levels were measured using a two-reagent, immunoturbidimetric system. The whole body scans including arms and legs, the trunk, and the head by using a Hologic Discovery A densitometer (Hologic, Inc., Bedford, Massachusetts), and the results of total BMD were obtained using Apex version 3.2 software. Moreover, age, gender, race, income-poverty ratio, body mass index (BMI), education level, alkaline phosphatase, cholesterol, calcium, phosphorus, globulin, and total protein were defined as the following covariates. Detailed measurement of HS-CRP and BMD and other covariate obtaining processes can be found at the website: www.cdc.gov/nchs/nhanes/.

### Statistical analyses

All estimates were derived based on the sample weights of the NHANES in accordance with the NCHS edited analysis guidelines, as the objective of NHANES is to generate data that is representative of the non-institutionalized civilian population of the USA. After adjusting for potential confounding factors, a multiple regression analysis was applied to assess the independent correlation between HS-CRP and total BMD. A smooth curve fitting was used to explain the nonlinear relationship of HS-CRP and total BMD. The subgroup analysis was performed using a weighted generalized additive model. Then, after adjusting for the same covariates in the linear regression models, a piece-wise linear regression model was conducted to reveal the threshold effect of HS-CRP on total BMD. All calculations were performed via R version 3.4.3 (http://www.R-project.org, The R Foundation) and Empower software (www.empowerstats.com; X&Y solutions, Inc., Boston MA).

### Ethics approval and consent to participate

The survey program was approved by the NCHS Institutional Review Board, and all participants had provided informed consent on their own or legally authorized representatives. All the methods were carried out in accordance with the relevant guidelines and regulations of NCHS Institutional Review Board.

## Results

### Baseline information of all participants

All of the participants’ weighted sociodemographic and medical characteristics were presented in Table 1. There were 1747 participants in this study. Among them, 47.1% were female, 29.4% were white, 19.5% were black, and 22.3% were Mexican–American. Among the four different groups of HS-CRP (quartiles, Q1–Q4), all comparisons of age, gender, BMI, income-poverty ratio, education level, alkaline phosphatase, cholesterol, calcium, phosphorus, globulin, total protein, and total BMD were significantly different except for race/ethnicity.

**Table 1:** Description of 1747 participants included in the present study.

HS-CRP	All	Q1	Q2	Q3	Q4	*P*-value
Age (years)	15.6 ± 2.4	15.0 ± 2.2	15.7 ± 2.4	16.0 ± 2.5	16.4 ± 2.6	< 0.0001
**Gender (%)**						
Male	52.9	58.0	55.9	54.1	43.8	0.0001
Female	47.1	42.0	44.1	45.9	56.2	
**Race (%)**						
White	29.4	54.5	54.3	53.3	50.2	0.2095
Black	19.5	13.4	12.5	10.2	11.3	
Mexican American	22.3	12.8	14.7	18.6	19.9	
Other	28.8	19.3	18.6	18.0	18.6	
BMI (kg/m^2^)	24.3 ± 6.0	20.5 ± 3.3	22.4 ± 4.0	24.5 ± 5.0	28.4 ± 7.2	< 0.0001
Income poverty ratio	2.3 ± 1.5	2.8 ± 1.5	2.6 ± 1.6	2.5 ± 1.6	2.5 ± 1.6	0.0022
**Education level (%)**						
Less than 9th	40.7	52.9	38.2	35.8	29.7	< 0.0001
9th–11th	54.1	42.9	55.2	53.4	56.5	
Other	5.2	4.2	6.5	10.8	13.8	
Alkaline phosphatase (U/L)	138.0 ± 96.3	164.2 ± 116.1	143.2 ± 99.5	130.4 ± 85.0	115.2 ± 78.4	< 0.0001
Cholesterol (mg/dL)	4.1 ± 0.76	4.0 ± 0.7	4.0 ± 0.7	4.2 ± 0.8	4.2 ± 0.8	< 0.0001
Calcium (mg/dL)	2.4 ± 0.1	2.4 ± 0.1	2.4 ± 0.1	2.4 ± 0.1	2.4 ± 0.1	< 0.0001
Phosphorus (mg/dL)	1.4 ± 0.2	1.4 ± 0.2	1.4 ± 0.2	1.3 ± 0.2	1.3 ± 0.2	< 0.0001
Globulin (g/L)	28.6 ± 3.8	25.9 ± 3.5	27.9 ± 3.4	28.7 ± 3.5	29.6 ± 3.7	< 0.0001
Total protein (g/L)	73.0 ± 4.0	71.8 ± 4.0	72.8 ± 4.0	72.7 ± 4.1	72.7 ± 4.1	0.0006
Total BMD (g/cm^2^)	1.0 ± 0.1	1.0 ± 0.1	1.0 ± 0.1	1.0 ± 0.1	1.1 ± 0.1	< 0.0001

Mean ± SD for continuous variables: P value was calculated by one-way ANOVA (normal distribution) and Kruskal–Wallis H (skewed distribution) test % for categorical variables: P value was calculated by chi-square test.

### Association between HS-CRP and total BMD

Four weighted univariate and multivariate linear regression models were constructed as follows: unadjusted model; adjusted for age, gender, and race as the minimally adjusted model^a^; adjusted for age, gender, race, BMI, income-poverty ratio, and education level as the minimally adjusted model^b^; and adjusted for the covariates in Table 1 as the fully adjusted model. A positive correlation between HS-CRP and BMD was found in unadjusted model (β = 0.007, 95% CI 0.003, 0.010). However, after adjusting the age, gender, and race in the minimally adjusted model^a^, this correlation was no longer significant (β = 0.001, 95% CI − 0.002, 0.004). Whereas, this correlation became negative again in the minimally adjusted model^b^ (β =  − 0.007, 95% CI − 0.009, − 0.004) and fully adjusted model (β =  − 0.006, 95% CI − 0.009, − 0.003), with both *P* for trend of < 0.05. Table 2 showed the detailed results.

**Table 2:** Association of HS-CRP with BMD.

	Unadjusted model β (95% CI)	Minimally adjusted model^a^ β (95% CI)	Partial adjusted model^b^ β (95% CI)	Fully adjusted model β (95% CI)
HS-CRP	0.007 (0.003, 0.010)	0.001 (− 0.002, 0.004)	− 0.007 (− 0.009, − 0.004)	− 0.006 (− 0.009, − 0.003)
P value	0.00009	0.37902	< 0.00001	0.00002
**HS-CRP (quartile)**				
Q1	Reference	Reference	Reference	Reference
Q2	0.039 (0.023, 0.055)	0.020 (0.007, 0.034)	0.010 (− 0.002, 0.023)	0.015 (0.002, 0.028)
Q3	0.047 (0.031, 0.063)	0.023 (0.009, 0.036)	0.005 (− 0.008, 0.017)	0.008 (− 0.006, 0.021)
Q4	0.057 (0.041, 0.073)	0.023 (0.010, 0.037)	− 0.014 (− 0.028, 0.000)	− 0.011 (− 0.026, 0.005)
*P* for trend	< 0.001	< 0.001	0.006	0.013

Unadjusted model adjust for: None.

Minimally adjusted model^a^ adjust for: age, gender, race.

Minimally adjusted model^b^ adjust for: age, gender, race, BMI, income-poverty ratio, education level.

Fully adjusted model adjust for: age, gender, race, BMI, income-poverty ratio, education level, alkaline phosphatase, cholesterol, calcium, phosphorus, globulin, and total protein.

**Figure 2:**
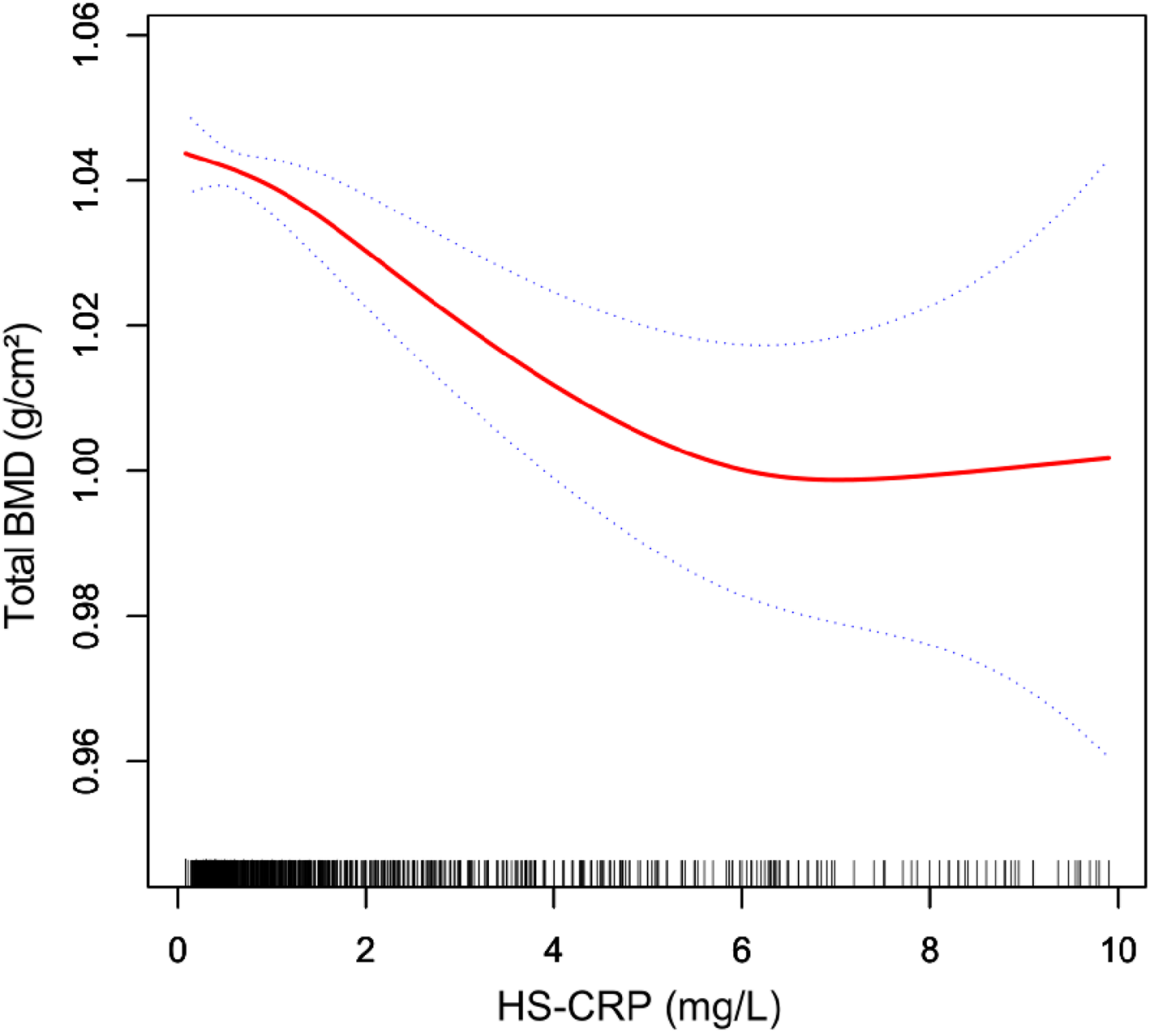
Correlation between HS-CRP and total BMD. Black points on the horizontal axis represent samples. The area between two blue dotted lines is expressed as a 95% CI. Each point shows the magnitude of the HS-CRP level and is connected to form a red continuous line. Age, gender, race, body mass index, income-poverty ratio, education level, alkaline phosphatase, cholesterol, calcium, phosphorus, globulin, and total protein were adjusted.

To further revealed the correlation between HS-CRP and total BMD, the generalized additive models and smooth curve fittings were conducted (Fig. 2). The adjusted smoothed graph indicated a non-linear association between HS-CRP and total BMD. As shown in Table 3, there were two turning points of 0.61 mg/L and 5.60 mg/L. A negative linear relationship was observed when HS-CRP ranged between 0.61 and 5.60 mg/L.

**Table 3:** Threshold effect analysis of HS-CRP on total bone mineral density using piece-wise linear regression.

Total bone mineral density	Adjusted ß (95% CI)	P-value
HS-CRP < 0.61 mg/L	0.020 (− 0.006, 0.046)	0.1398
0.61 mg/L ≤ HS-CRP ≤ 5.60 mg/L	− 0.010 (− 0.015, − 0.004)	0.0002
HS-CRP > 5.60 mg/L	0.008 (− 0.009, 0.025)	0.3467

Age, gender, race, BMI, income-poverty ratio, education level, alkaline phosphatase, cholesterol, calcium, phosphorus, globulin, and total protein were adjusted.

Finally, we conducted subgroup analyses for stratifying by age and gender (Table 4). In unadjusted model, a positive association between HS-CRP and total BMD was observed when stratifying by gender (male: β = 0.007, 95% CI 0.002, 0.013; and female: β = 0.007, 95% CI 0.003, 0.011). In minimally adjusted model, only adolescents aged 15–20 years had a positive association between HS-CRP and total BMD (β =  − 0.004, 95% CI 0.001, 0.008). In the fully adjusted model, all subgroup analyses indicated a negative association between HS-CRP and total BMD (10–14 years: β =  − 0.006, 95% CI − 0.011, − 0.002; 15–20 years: β =  − 0.005, 95% CI − 0.009, -0.001; male: β =  − 0.006, 95% CI − 0.010, − 0.001; and female: β =  − 0.006, 95% CI − 0.010, − 0.003).

**Table 4:** Association between HS-CRP (mg/L) and total BMD (g/cm^2^), stratified by age and sex.

	Unadjusted model β (95% CI)	Minimally adjusted model β (95% CI)	Fully adjusted model β (95% CI)
**Stratified by age**			
10–14 years	0.003 (− 0.002, 0.008)	0.002 (− 0.003, 0.007)	− 0.006 (− 0.011, − 0.002)**
15–20 years	0.003 (− 0.001, 0.006)	0.004 (0.001, 0.008)*	− 0.005 (− 0.009, − 0.001)**
**Stratified by gender**			
Male	0.007 (0.002, 0.013)*	0.002 (− 0.002, 0.006)	− 0.006 (− 0.010, − 0.001)*
Female	0.007 (0.003, 0.011)***	0.002 (− 0.001, 0.006)	− 0.006 (− 0.010, − 0.003)***

Unadjusted model adjust for: None.

Minimally adjusted model adjust for: age, gender, race.

Fully adjusted model adjust for: age, gender, race, BMI, income-poverty ratio, education level, alkaline phosphatase, cholesterol, calcium, phosphorus, globulin, and total protein.

Gender is not adjusted in the subgroup analysis stratified by gender, and age is not adjusted in the subgroup analysis stratified by age. *P < 0.05, **P < 0.01, ***P < 0.001.

## Discussion

Our cross-sectional study here first investigated whether HS-CRP was independently related to total BMD in 10–20 years old adolescents via a large and nationally representative sample from the NHANES database. Our results mainly indicated that HS-CRP was negatively associated with total BMD in 10–20 years old adolescents. In addition, the independent and negative association was also observed in subgroup analyses regarding age and gender.

Previous studies had investigated the correlation between CRP and bone health. Some previous studies had similar findings to our results that inflammatory markers, such as CRP, had a negative relationship with BMD. A cross-sectional study performed by Koh et al*.* showed that higher CRP levels in women were associated with lower BMD^9^. Ding et al*.* demonstrated that CRP levels were negatively associated with changes in total body BMD in a longitudinal study^8^. In addition, higher CRP levels had been shown to be related to increased markers of bone turnover^12^. However, a study by Menezes et al*.* showed that CRP levels had no relationship with BMD or plasma osteocalcin levels, which is a bone turnover marker^13^. Berglundh et al*.* also demonstrated that CRP was not an indication of bone loss, low BMD, or fracture in older women^14^. Considering that there were only a few studies on this issue, thus differences in the demographic base, design protocols, number of participants, and controlled confounders of these studies were bound to result in controversial and contradictory findings.

Till now, there is very limited evidence linking HS-CRP and BMD in adolescents. A recent cross-sectional study of 5249 in a young adult birth cohort that found a CRP showed a negative association with total BMD in females but no association in males^13^. Their findings were partially consistent with the results of our subgroup analysis. This could be attributed to that we focused on the HS-CRP but not traditional CRP. For that traditional CRP ranges from 10 to 1000 mg/L while HS-CRP can be accurate to less than 10 mg/L. In particular, an HS-CRP above 10 mg/L is considered a stressful condition and excluded in our study. Our results are therefore more precise and can be used to describe the correlation between HS-CRP and BMD in normal state adolescents.

Following the STROBE guideline^15^, we not only performed piece-wise linear regression but also conducted subgroup analyses to better understand the correlation between HS-CRP and BMD. As a result, we observed two significant turning points (0.61 mg/L and 5.60 mg/L). Their total BMD decreases with elevated HS-CRP among the turning points. We also observed that this negative association was still significant in 10–14 years, 15–20 years, male, and female adolescents in our subgroup analyses. However, further prospective studies with large samples were warranted to support this conclusive finding.

The underlying relationship between HS-CRP and bone remodeling is still uncertain. A possible explanation of the negative effect of CRP on bone formation may be due to that an increased level of inflammatory cytokines, such as IL-1, IL-6, and TNF-α induces an imbalance of the RANK/RANKL/OPG system, known for regulating the differentiation of osteoclasts and osteoblasts to reduce the total BMD^16^. CRP plays a key role in the inflammatory process by binding to Fc receptors to induce the release and elevated activity of pro-inflammatory cytokines such as IL-6^17,18^. Therefore, HS-CRP may be involved in the pathogenesis of abnormal bone metabolism via IL-6 indirectly. These results indicated that HS-CRP had the capacity to be an indicator of BMD.

A potential strength of our study is the large representative sample of multi-racial populations to better generalize to the US population. Such a large sample size made it possible to conduct a detailed assessment of potential confounders. Furthermore, we used the more accurate HS-CRP rather than the traditional CRP used in previous studies, which makes our study more convincing.

However, our study also had some shortcomings. First, whether there was a relationship of causality between HS-CRP and total BMD was hard to be determined through a cross-sectional design study. Second, other inflammatory markers, such as TNF-α and IL-6, may provide useful additional information but data were not available. Thus, our statistical findings on the relationship between HSCRP and BMD need to be confirmed in the intervention trial with large sample size.

## Conclusions

In summary, this study revealed a negative correlation between HS-CRP level and total BMD in adolescents aged 10–20 years. In particular, BMD decreased with increasing HS-CRP levels among the turn points of turning points 0.61 mg/L and 5.60 mg/L. This negative correlation was also observed in age and gender stratification. The impact of HS-CRP on adolescent bone health needs further and more detailed investigations in larger cohort studies to investigate the consistency and reproducibility of our findings. Biological studies are also needed to survey the impact of HS-CRP on bone metabolism to investigate the biological rationality of the observed negative correlation.

## Data Availability

The data presented in this study are available on the NHANES. Methodological details about the NHANES are available at www.cdc.gov/nchs/nhanes/.
